# SNP Diversity and Genetic Structure of “Rogosija”, an Old Western Balkan Durum Wheat Collection

**DOI:** 10.3390/plants12051157

**Published:** 2023-03-03

**Authors:** Ana Velimirović, Zoran Jovović, Dragan Perović, Heike Lehnert, Sanja Mikić, Dragan Mandić, Novo Pržulj, Giacomo Mangini, Mariella Matilde Finetti-Sialer

**Affiliations:** 1 Biotechnical Faculty Podgorica, University of Montenegro, Mihaila Lalića 15, 81000 Podgorica, Montenegro; 2 Federal Research Centre for Cultivated Plants, Institute for Resistance Research and Stress Tolerance, Julius Kuehn-Institute, Erwin-Baur-Strasse 27, 06484 Quedlinburg, Germany; 3 Federal Research Centre for Cultivated Plants, Institute for Biosafety in Plant Biotechnology, Julius Kuehn-Institute, Erwin-Baur-Strasse 27, 06484 Quedlinburg, Germany; 4 Institute of Field and Vegetable Crops, Maksima Gorkog 30, 21101 Novi Sad, Serbia; 5 Agricultural Institute of Republika Srpska, Knjaza Miloša 17, 78000 Banja Luka, Republika Srpska, Bosnia and Herzegovina; 6 Faculty of Agriculture, University of Banjaluka, Bulevar vojvode P. Bojovića 1a, 78000 Banja Luka, Republika Srpska, Bosnia and Herzegovina; 7 Institute of Biosciences and Bioresources, National Research Council (IBBR-CNR), Via Amendola 165/A, 70126 Bari, Italy

**Keywords:** durum wheat landraces, Rogosija, genetic diversity, UPOV descriptors, 25K Illumina SNP array, Western Balkan eco-geographic region

## Abstract

Durum wheat landraces represent a genetic resource for the identification and isolation of new valuable genes and alleles, useful to increase the crop adaptability to climate change. Several durum wheat landraces, all denominated “Rogosija”, were extensively cultivated in the Western Balkan Peninsula until the first half of the 20th century. Within the conservation program of the Montenegro Plant Gene Bank, these landraces were collected, but without being characterized. The main goal of this study was to estimate the genetic diversity of the “Rogosija collection” consisting of 89 durum accessions, using 17 morphological descriptors and the 25K Illumina single nucleotide polymorphism (SNP) array. The genetic structure analysis of the Rogosija collection showed two distinguished clusters localized in two different Montenegro eco-geographic micro-areas, characterized by continental Mediterranean climate and maritime Mediterranean climate. Data suggest that these clusters could be composed of two different Balkan durum landrace collections evolved in two different eco-geographic micro-areas. Moreover, the origin of Balkan durum landraces is discussed.

## 1. Introduction

Wheat cultivation accounts for nearly 220 million ha in the world [1]. The species with significant agricultural importance are common wheat (*Triticum aestivum* L., 2n = 6x = 42, BBAADD) and durum wheat (*Triticum turgidum* L. subsp. *durum* (Desf.), 2n = 4x = 28, BBAA). Historically, the cultivation of wheat consisted in the sowing of diverse plant assemblages to lower the risks of crop failure and increase food security, because traditional farmers had a limited capacity to control spatially heterogeneous and temporally unpredictable environments [2]. This rural practice led to the development of different wheat landraces, maintained by traditional farmers who played a fundamental role conserving the heritage of local germplasm for generations. The landraces kept in traditional farming systems represent a germplasm reservoir that deserves attention by owning a broad genetic diversity. Indeed, the landraces evolved in restricted areas are well adapted to local climatic and edaphic environments. This is due to specific phenotypic plasticity caused by genotypic variation. The durum landraces are a genetic resource where it is possible to detect alleles related to abiotic and biotic tolerance, grain quality (protein, antioxidant compounds, and mineral elements), and adaptation to low-input cropping systems. These alleles can be exploited in breeding programs to broaden the genetic basis of durum wheat cultivars [3].

Wheat was introduced into the Balkan Peninsula through human migrations from Anatolia in the Neolithic era. Charred remains of einkorn wheat (*Triticum monococcum* L.) dating into the early VI millennium BC have been found in Serbia [4]. In the following ages, the cultivation of tetraploid wheats, such as landraces of emmer (*Triticum turgidum* subsp. *dicoccum* (Schrank ex Schübler) Thell.), durum, and turgidum (*Triticum turgidum* subsp. *turgidum*) was largely extended to the Balkan Peninsula. The territory near Podgorica (Montenegro), especially, was known for the cultivation of durum landraces, all named “Rogosija” [5,6]. This name probably originates from long awns, similar to animal horns (a local term for horn is “rog”) [7].

Starting from 1700, in the Northern Balkan regions, the Rogosija landraces were gradually replaced by common wheat landraces [8]. After the Second World War, the common wheat cultivars obtained from breeding programs completely ousted the common wheat landraces. Nevertheless, in Montenegro, Bosnia, Herzegovina, and south-eastern Serbia, the Rogosija landraces were cultivated until the mid-1970s [9]. From that time onwards, many farms discontinued the cultivation of tetraploid wheat landraces and directly replaced them with high-yielding common cultivars. [10].

Therefore, intensive activities on collecting durum accessions were started in Montenegro, with the aim to preserve the Balkan durum landraces. A large “Rogosija collection”, consisting of 125 durum accessions, was collected in Montenegro, Croatia, and Bosnia and Herzegovina in the period from 1955 to 1964. As of the year 2000, the financial resources of the conservation program were reduced, which caused a partial loss of the collection. The remaining Rogosija collection consisting of 89 accessions stored in the Montenegro Plant Gene Bank was regenerated at Danilovgrad (Montenegro) during the vegetation season 2018–2019.

The use of molecular methods to assess genetic diversity proved to be consistently useful for a correct classification of different genotypes and for the identification of duplicates, quite often present in gene bank collections [11,12,13]. The availability of single-nucleotide polymorphism (SNP) markers generated by next-generation sequencing (NGS) and the release of the durum wheat reference genome, allowed understanding the genetic structure of durum collections, including cultivars and landraces [14,15,16,17].

The aims of the present research were (1) to study the genomic diversity and population structure of the Rogosija collection deposited in the Montenegro Plant Gene Bank by using morphological and SNP markers, and (2) to investigate correlation between genetic clusters and eco-geographic conditions of the Western Balkan Peninsula.

## 2. Results

### 2.1. Morphological Diversity in Western Balkan Durum Accessions

**Figure 1:**
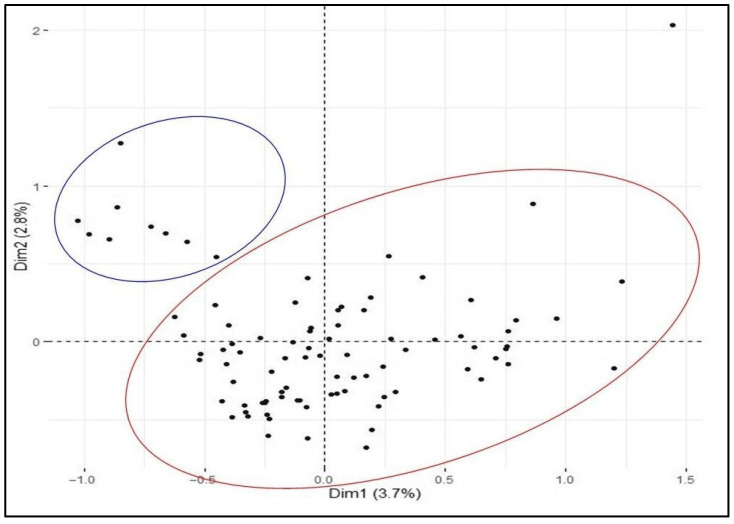
Multiple Correspondence Analysis (MCA) of 89 Western Balkan durum accessions, based on 17 morphological descriptors. Dim1 = dimension 1, and Dim2 = dimension 2. Cluster A and Cluster B are circled in red and blue, respectively.

Multiple Correspondence Analysis (MCA) based on 17 morphological descriptors split the Rogosija collection in two clusters which included 79 (Cluster A) and 9 (Cluster B) durum accessions (Figure 1). The first two dimensions explain 3.7% and 2.8% of the variation. The Cluster B grouped durum accessions show a stronger curvature of beak (assigned as 7) than the Cluster A (assigned as 1). In addition, the two clusters resulted in contrasting recurved flag leaves, ear length, and lower glume descriptors such as length of beak and width of shoulder (Table S1). The durum accession METD-5/02, characterized by white ear and brown awns, resulted outside both clusters.

### 2.2. SNP Diversity in Western Balkan Durum Accessions

The Rogosija collection and the four additional foreign durum cultivars were analyzed with a high-throughput genotyping system based on the 25K Illumina SNP wheat array. A total of 6915 high-quality SNPs were retained after filtering and mapped on the 14 chromosomes of the durum genome [18]. Identity by State (IBS) values ranged between 0.46 and 1.00 (Figure S1). A total of 105 pairs of IBS values had results higher than 0.95, suggesting the presence of duplicates and durum accessions very closely related in the Rogosija collection. Among the pairs, a durum accession (METD 18/03) showed a high IBS value with the durum reference Taganrog. According to the IBS threshold values (>0.95), 42 Western Balkan durum accessions were identified and split into 13 identity groups with high similarity (Table S2). For each identity group, a single durum accession was included in the diversity analysis. In this way, 60 Western Balkan durum accessions of the Rogosija collection and four additional durum cultivars (used as references) were included in the analysis.

**Figure 2:**
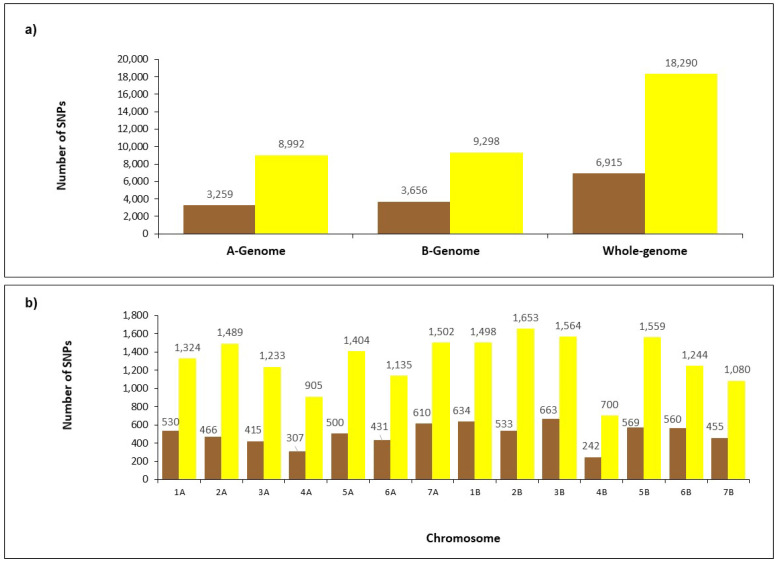
Distribution of SNPs generated from the 25K Illumina array across the genome (**a**) and chromosome (**b**) in the Rogosija collection, including 60 Western Balkan durum accessions and four foreign durum cultivars. Total and polymorphic SNPs are shown in yellow and brown colors, respectively.

The SNP markers were unequally distributed across genomes and chromosomes. A higher number of polymorphic SNPs was recorded on the B genome (3656) than the A genome (3259) (Figure 2a). The highest number of SNPs was recorded on chromosome 2B (1653), while the smallest was found on chromosome 4B (700) (Figure 2b). Chromosome 4B showed the smallest number of polymorphic SNPs (242), while the highest was found on chromosome 3B (663). A high polymorphism level was found on chromosome 3B, when comparing the number of SNPs between the Rogosija collection and the foreign durum cultivars. Both groups showed a low SNP number on chromosome 4B (Figure S2).

**Figure 3:**
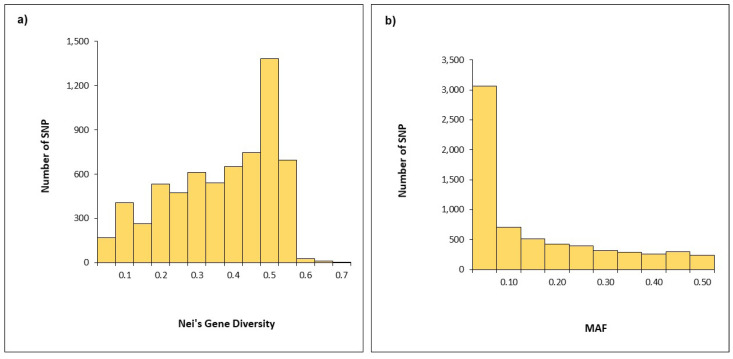
Frequency distribution of Nei’s gene diversity values (**a**) and minor allelic frequency (MAF) (**b**) for 6915 polymorphic SNPs in the Rogosija collection, including 60 Western Balkan durum accessions and four foreign durum cultivars.

Nei’s gene diversity of SNPs ranged from 0.01 to 0.60 (Figure 3a) and over the half of the SNPs showed minor allele values (MAF) lower of 0.10 (Figure 3a,b). Nei’s gene diversity ranged from 0.313 (chromosome 5B) to 0.368 (chromosome 3B), while MAF spanned from 0.116 (chromosome 7A) to 0.159 (chromosome 4B) (Table 1). Interestingly, the chromosomes 1A, 4A, 1B, and 6B showed a Nei’s gene diversity and MAF slightly higher than the average values of the whole genome (Nei’s gene diversity = 0.341 and MAF = 0.138). The B genome showed higher values of Nei’s gene diversity and MAF than the A genome.

**Figure 4:**
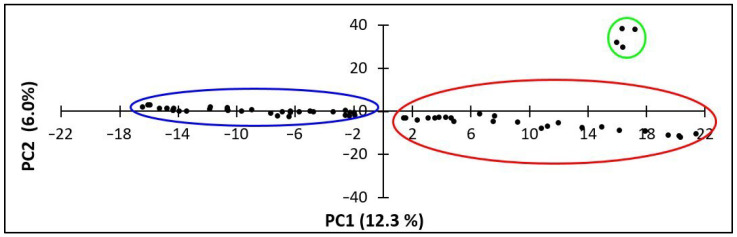
Principal Component Analysis (PCA) of the Rogosija collection, including 60 Western Balkan durum accessions and four foreign durum cultivars. The two genetic clusters of Western Balkan accessions are circled in blue and red colors; the cluster of foreign durum cultivars is circled in green.

The Principal Components Analysis (PCA) showed three different genetic durum clusters (Figure 4). In particular, the Rogosija collection was split in two clusters: Cluster 1 (including 25 durum accessions) shown in the second and third quadrant, and Cluster 2 (including 35 durum accessions) spread in the fourth one. The foreign durum cultivars, used as references, constituted a distinguished cluster from the Rogosija clusters, suggesting no genetic relationship with the Western Balkan germplasm.

**Figure 5:**
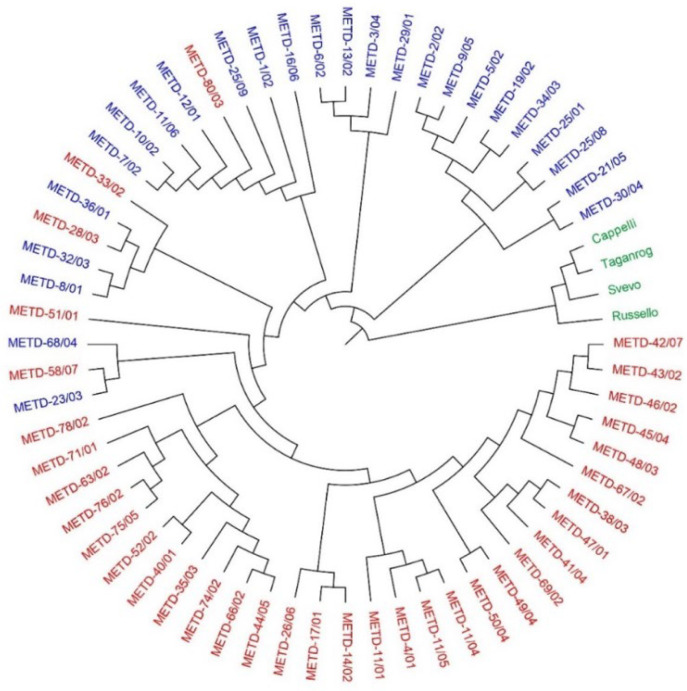
Unrooted UPGMA tree of genetic distances for the Rogosija collection, including 60 Western Balkan durum accessions and four foreign durum cultivars, based on 6915 SNPs. The two clusters of Balkan accessions are reported in blue and red colors; the cluster of foreign durum cultivars is reported in green.

The phylogenetic tree was consistent with the PCA results. The Rogosija collection was split in two clusters, separated from the foreign durum cultivars (Figure 5).

The Analysis of Molecular Variance (AMOVA) revealed that 16% of the total variation was due to differences among the clusters, whereas the remaining (84%) variance was within clusters (Table 2). The genetic diversity of each of the two Western Balkan durum clusters (obtained by PCA) was estimated (Table 3). Cluster 1 showed the lowest number of effective alleles per locus (*Ne*) (1.443), Shannon’s information index (*I*) (0.416), diversity index (*h*) (0.266), and percentage of polymorphic loci (*PPL*) (88.24%). Cluster 2 showed the highest values for all indices (*Ne* = 1.577, *I* = 0.510, *h* = 0.321, and *PPL* = 88.55%).

### 2.3. Relationships between Rogosija Durum Clusters and Eco-geographic Conditions

**Figure 6:**
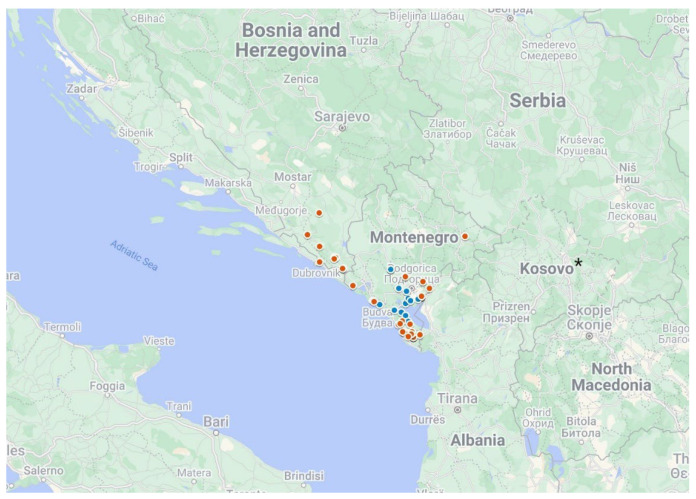
Geographic sampling sites of the durum accessions collected in the Western Balkan Peninsula [19]. The accessions included in Cluster 1 and Cluster 2 are shown as blue and red dots, respectively. * References to Kosovo shall be understood to be in the context of Security Council Resolution 1244 (1999).

The geographic positions of the Western Balkan durum accessions, split according to the genetic clusters defined by polymorphic SNPs, are reported in Figure 6. A spatial separation was observed; Cluster 1 was mapped in the continental Montenegrin regions, and Cluster 2 localized in the Western Balkan Coastal region of Montenegro, Bosnia, Herzegovina, and Croatia [19].

A total of 51 out of 60 durum accessions analyzed in this study were localized in Montenegro; there were 24 mapped around Skadar Lake and 27 in the coastal area. Therefore, the 51 Montenegrin durum accessions were used to explore relationships between the two genetic clusters and climatic regions. According to the Köppen climatic classification [20], the Montenegrin durum accessions were included in the two climatic regions named Csa and Cfsb (Figure S3). The Csa climatic region is characterized by a moderately warm, rainy climate with hot summers and a pronounced summer dry period. The Cfsb climatic region shows a moderately warm, rainy climate with warm summers but no pronounced dry period during the year. The climatic region Csa comprised over the 85% of Montenegrin durum samples. Interestingly, in this climatic region, the Montenegrin durum accessions, split according to the genetic clustering results, were localized in two different eco-geographic micro-areas. The first Montenegrin eco-geographic micro-area, located around Skadar Lake, is characterized by a continental Mediterranean climate with a very long and warm, dry summer period, with high temperatures often between 35 °C and 40 °C. The second Montenegrin eco-geographic micro-area located in the coastal region, has a maritime Mediterranean climate, with wet, rainy, and mild winters and warm and dry summers (Table S3).

## 3. Discussion

Durum wheat landraces, collectively known as Rogosija, were the main cereal of Western Balkan Peninsula until mid-20th century, covering over 80% of arable land of the Montenegro and Herzegovina littoral zone [21]. After the Second World War, the introduction of high-yielding, winter common wheat cultivars threatened the survival of Rogosija landraces, when almost all disappeared from farmers’ fields in Montenegro and Herzegovina [21,22]. Starting from 1955, sampling of durum wheat accessions in Montenegro allowed the conservation of the Rogosija collection in the Montenegro Plant Gene Bank for the next 55 years [23]. This collection represents an unexplored durum wheat germplasm that can be analyzed for genetic diversity. In addition, the Rogosija collection conserved in Montenegro Plant Gene Bank showed a relationship with the eco-geographic profiles of the sampling regions.

The common tools used for diversity studies of plant genetic resources are morphological markers [24,25]. They provide valuable information for characterization of collections stored in gene banks [26]. The main morphological markers used in wheat are based on the UPOV descriptors [17,27,28,29]. In the present study, a panel of 89 Western Balkan durum accessions was initially evaluated with morphological descriptors. Cluster analysis identified two groups, differing in recurved flag leaves and beak curvature. The discriminative power of UPOV descriptors to differentiate the Balkan durum germplasm was validated by Takač and colleagues [30] in a Serbian durum collection including cultivars and breeding lines. According to our results, the two clusters showed a narrow morphological diversity as observed by the low cumulative variance value (<10%) of the first two dimensions of MCA.

Single nucleotide polymorphism (SNP) markers have become fundamental for both genetic studies and breeding programs. In addition, the development of wheat high-density SNP array provided the most innovative tool for genetic diversity and the population structure estimation in durum collections, including cultivars and landraces [17,31,32,33,34,35]. Therefore, the 25K SNP array was used to assay the SNP diversity in the Rogosija collection and in four foreign durum cultivars used as references. Over 6900 polymorphic SNPs were found, mostly mapped on genome B, as observed in other durum collections [31,32,33,34]. The Nei’s diversity indices of the Rogosija collection were slightly lower than the Nei’s diversity index observed by Mazzucotelli et al. [33] in the global durum collection, confirming the narrow genetic variability observed with the UPOV descriptors.

According to the IBS analysis, we discarded 29 Western Balkan durum accessions considered duplicated and/or synonymous. This is not surprising because historically a traditional exchange of seeds occurred between Balkan farmers. Moreover, our result confirms that the SNP markers are an efficient tool to identify duplicate accessions stored in gene banks [32], especially when morphological markers fail.

The 6915 high quality markers generated by SNP genotyping were suitable to define the genetic structure of the Rogosija collection. The PCA and phylogenetic analyses revealed three clusters: two distinguished clusters from the Western Balkan durum accessions, and a third cluster comprising foreign durum cultivars (Cappelli, Taganrog, Russello, and Svevo). This result confirms the reliability of the applied analytical approach, suggesting that the durum germplasm known as Rogosija and stored in Montenegro Plant Gene Bank likely consists of two different durum landrace groups, with no relation to the Italian durum cultivars (Cappelli, Russello, and Svevo). Considering the maritime trades between the Italian and Balkan Peninsulas, it was suggested that Balkan durum landraces had an Italian origin [36]. Indeed, historical evidence has shown that Italian durum germplasm was introduced in the Balkan Peninsula during the Second World War, but only the durum cultivar Cappelli was spread in the Western Balkan regions [37]. In our study, no relation between the durum cultivar Cappelli and the Western Balkan durum accessions was found, indicating a different origin of the Balkan durum germplasm as suggested by Pavićević [37]. Our results indicate that the Balkan durum germplasm might have its origin in the Eastern regions. One durum wheat accession was a duplicate of Taganrog, an old Russian landrace [38]. Nazco et al. [39] observed that the durum landraces from the Northern Balkans were very different in quality traits when compared with cultivars from Eastern and Western Mediterranean countries, suggesting a different origin. This assumption was supported by Dedkova et al. [40], who demonstrated that emmer accessions from former Yugoslavia, Bulgaria, and Russia do not carry the 7A:6B translocation, which is common in the emmer accessions from Western Mediterranean countries. Hence, these authors proposed a division of European emmer into two groups: West European and Volga-Balkan.

The AMOVA analysis revealed a higher genetic variance within, rather than among clusters of Western Balkan accessions, suggesting high and frequent rates of seed trade off among farmers in this area.

To estimate whether the genetic clusters detected are related to the Balkan eco-geographic regions, the durum accessions were geo-referenced and evaluated according to the ecological data of the Rogosija collecting sites. Interestingly, 24 Montenegrin durum accessions contained in the Cluster A were collected around Lake Skadar, while 27 included in the Cluster B were sampled in the Montenegrin littoral coast. Continental Mediterranean climate and maritime Mediterranean clime are the ecological conditions of Lake Skadar zone and littoral coast, respectively. This result supported that the Rogosija collection stored in the Montenegro Plant Gene Bank enclosed two different landraces, evolved in two different eco-geographic micro-areas. Similar results were observed in a Tunisian durum collection, where the genetic clusters showed a strong genetic stratification from the north to the south of Tunisia site [41]. A large *T*. *turgidum* subsp. *dicoccum* collection representing a wide geographic range of emmer accessions, using DArTseq was split into four distinguished clusters in accordance with their eco-geographic origin [42]. Relationships between genetic clusters and eco-geographic areas of the Balkan Peninsula were also found in other species such as grapevine [43], lentil [44], and dill [45] confirming the key role of this region for the biodiversity.

## 4. Materials and Methods

### 4.1. Plant Material and Morphological Trait Characterization

The Rogosija collection, including 89 Western Balkan durum accessions provided by the Montenegro Plant Gene Bank, was analyzed. The experiment was carried out in the 2020–2021 season at the Research Unit in Danilovgrad (Montenegro). Twenty seeds of each durum accession were sown at 5 cm depth in plots consisting of 1 m rows, 60 cm apart. During the growing season, 10 g of nitrogen per m^2^ and standard cultivation practices were applied. According to guidelines by the International Union for Protection of New Varieties of Plants [46], a panel of 16 morphological descriptors was used for phenotypic characterization of the accessions (Table S1). In addition, plant height was evaluated using the following scale: 1 ≤114 cm; 3 = 115–129 cm; 5 = 130–144 cm; 7 = 145–159; 9 ≥ 160 cm. Data were scored on ten plants, harvesting a random sample of representative spikes. The data were used to obtain morphological cluster, based on the Multiple Correspondence Analysis (MCA), in R package FactoMineR v.2.7 (http://factominer.free.fr/ (accessed on 2 March 2023)).

### 4.2. DNA Extraction and SNP Genotyping

The Rogosija collection and four durum cultivars (Cappelli, Russello, Taganrog, and Svevo), provided by the Institute of Biosciences and Bioresources, National Research Council (Bari, Italy), were genotyped. Seeds collected of each accession were germinated in jiffy pots and grown under controlled conditions (20 °C day/16 °C night temperature, 16 h light/8 h dark photoperiod and 70% relative humidity). Genomic DNA was extracted from 100 mg of fresh leaves of a bulk of 10-day-old seedlings using DNeasy™ Plant Mini Kit (Qiagen, Hilden, Germany) following the manufacturer’s instructions. Genomic DNA quality was checked using 1% agarose gel electrophoresis. The DNA of each sample was used for SNP genotyping using an optimized wheat 25K Infinium iSelect array (Illumina Inc., San Diego, CA, USA). The array contains 24,145 SNPs combining 17,229 markers from the Illumina 20K Illumina Infinium array [47], 6916 new markers from the 135K Axiom array [48], and additional trait- and gene-specific markers. Genotyping was performed by the SGS Institute Fresenius, Trait Genetics GmbH (Gatersleben, Germany) with the GenomeStudio v.2.0 software package (Illumina, San Diego, CA, USA).

### 4.3. Genetic Diversity Analysis

Physical SNP positions were obtained by alignment of the 25K array design file to the reference genome of the durum wheat cultivar Svevo [18]. The SNPs on unlinked chromosomes were discarded. Monomorphic SNPs, and those with more than 10% missing values were excluded. SNP markers were filtered for Minor Allele Frequency (MAF), and values less than 5% were excluded from analysis. In this way, a panel of high-quality SNPs were used to calculate Identity by State (IBS) similarities and to identify putative durum duplicates accessions (IBS > 0.99) and very strongly related (IBS > 0.95) accessions. Furthermore, to explore the genetic variability the SNP panel was used to perform Principal Component Analysis (PCA) [49] and phylogenetic analysis using the Neighbor Joining clustering method [50]. SNP filtering, IBS, and clustering analyses were carried out using the TASSEL software v.5.0 [51]. The resulting tree was displayed using FigTree v. 1.4.3 [52]. The Analysis of MOlecular VAriance (AMOVA) was performed to check the significance of the variance between groups obtained from cluster analysis. The genetic indices number of effective alleles (*Ne*), Shannon’s information index (*I*), heterozygosity observed (*h*) and percentage of polymorphic loci (*PPL*) were estimated using GenAlex v.6.5 software [53].

### 4.4. Eco-Geographic Profile of the Germplasm Collection

The geographic coordinates were taken for all accessions as shown on Google maps [18]. The climatic data were retrieved from meteorological stations in the areas of their cultivation. Since the last evidence of fields under durum wheat in this area was in 1972 [9], data refer to the period from 1950 to 1975 [54]. Climate data included 30 traits related to temperature, insolation, precipitation, and tropical days as reported in Table S3. The Institute for Hydrometeorology and Seismology of Montenegro kindly provided all data [54].

## 5. Conclusions

This study showed morphological and SNP diversity in the Western Balkan accessions. In the Rogosija collection, the SNPs represent a powerful tool to study the genetic structure of wheat collections, and to detect duplicate or redundant durum accessions in comparison to morphological descriptors. These results suggest that the SNPs might be useful to define efficient strategies for the conservation of durum germplasm in gene banks.

According to SNP genotyping, the old Balkan durum collection Rogosija was split into two distinguished clusters related to two different eco-geographic micro-areas of Montenegro. These results indicated that the Rogosija collection likely consists of two different durum wheat landraces.

The genetic diversity observed in the durum Rogosija collection deserves further investigations. The durum Rogosija accessions can be included in association mapping studies to identify new alleles to contrast the effect of climate change. Finally, accurate phenotyping assays could be performed to select accessions useful for durum breeding programs.

## Figures and Tables

**Table 1:** Mean Nei’s gene diversity and minor allelic frequency (MAF) per genome and chromosome for 6915 polymorphic SNPs in the Rogosija collection, including 60 Western Balkan durum accessions and four foreign durum cultivars.

Chromosome	Nei’s Gene Diversity	MAF
1A	0.361	0.149
2A	0.338	0.136
3A	0.327	0.136
4A	0.353	0.155
5A	0.328	0.118
6A	0.331	0.133
7A	0.328	0.117
A-Genome	0.338	0.135
1B	0.345	0.149
2B	0.352	0.136
3B	0.368	0.134
4B	0.339	0.159
5B	0.313	0.136
6B	0.355	0.157
7B	0.331	0.116
B-Genome	0.343	0.141
Whole Genome	0.341	0.138

**Table 2:** Analysis of molecular variance (AMOVA) of 60 western Balkan durum accessions (Rogosija collection), split according to genetic structure estimated using 6915 polymorphic SNPs.

Source of Variation	df	SS	MS	Est. Var.	%	*p* Values
Among clusters	1	6722.42	6722.42	194.23	16%	<0.001
Within cluster	58	61,333.55	1057.47	1057.47	84%	
Total	59	68,055.97		1251.70	100%	

Abbreviations: df = degree of freedom, SS = sum of squares, MS = mean squares, Est. Var. = estimate of variance, % = percentage of total variation.

**Table 3:** Number of effective alleles (*Ne*), Shannon’s information index (*I*), observed heterozygosity (*h*), and percent of polymorphic loci (*PPL*), for 60 western Balkan durum accessions (Rogosija collection) according to genetic structure, estimated using 6915 polymorphic SNPs.

Western Balkan Cluster	Number of Accession	*Ne*	*I*	*h*	*PPL*
1	25	1.443	0.416	0.266	88.24
2	35	1.577	0.510	0.321	88.55

## Data Availability

Data are contained within the article or supplementary material.

## Supplementary Materials

The following supporting information can be downloaded at https://www.mdpi.com/article/10.3390/plants12051157/s1, Figure S1: Box plot of Identity by State (IBS) in the Rogosija collection including 89 durum accessions determined by the analysis of 6915 SNP markers; Figure S2: Distribution of SNPs generated from the 25K Illumina SNPs array across the chromosome in the Rogosija collection (in purple) and in the foreign durum cultivars (green); Figure S3: Köppen climatic classification of Montenegro [54]. Csa = hot summer Mediterranean climate; Cfsb = perhumid Mediterranean mountain climate without summer dryness; Cfs’’b = moderately warm Mediterranean climate without pronounced dry period during the year with dry summers; Cfb = temperate oceanic climate; Cfwas’’Bx’’ = moderately temperate climate without pronounced dry period during the year with less precipitation in the winter part of the year, and Dfbx’’ = warm summer humid continental climate; Table S1: Morphological descriptors of the Rogosija collection including 89 Western Balkan durum accessions; Table S2: List of Identity groups, and accessions showing Identity by State (IBS) > 0.95. For each Identity group a single accession was considered in phylogenetic analysis; Table S3: Eco-geographical data of two micro-areas identified in the Montenegrin CSA climatic region where the Rogosija collection was sampled.
