# Supplementary material for: SNP Diversity and Genetic Structure of “Rogosija”, an Old Western Balkan Durum Wheat Collection
Source: Plants (Basel). 2023 Mar 3;12(5):1157. doi: 10.3390/plants12051157 (PMC10005349; doi:10.3390/plants12051157)
Supplement: Supplementary file 1 [file plants-12-01157-s001.zip › Supplementary Figures.pptx]

## Slide 1
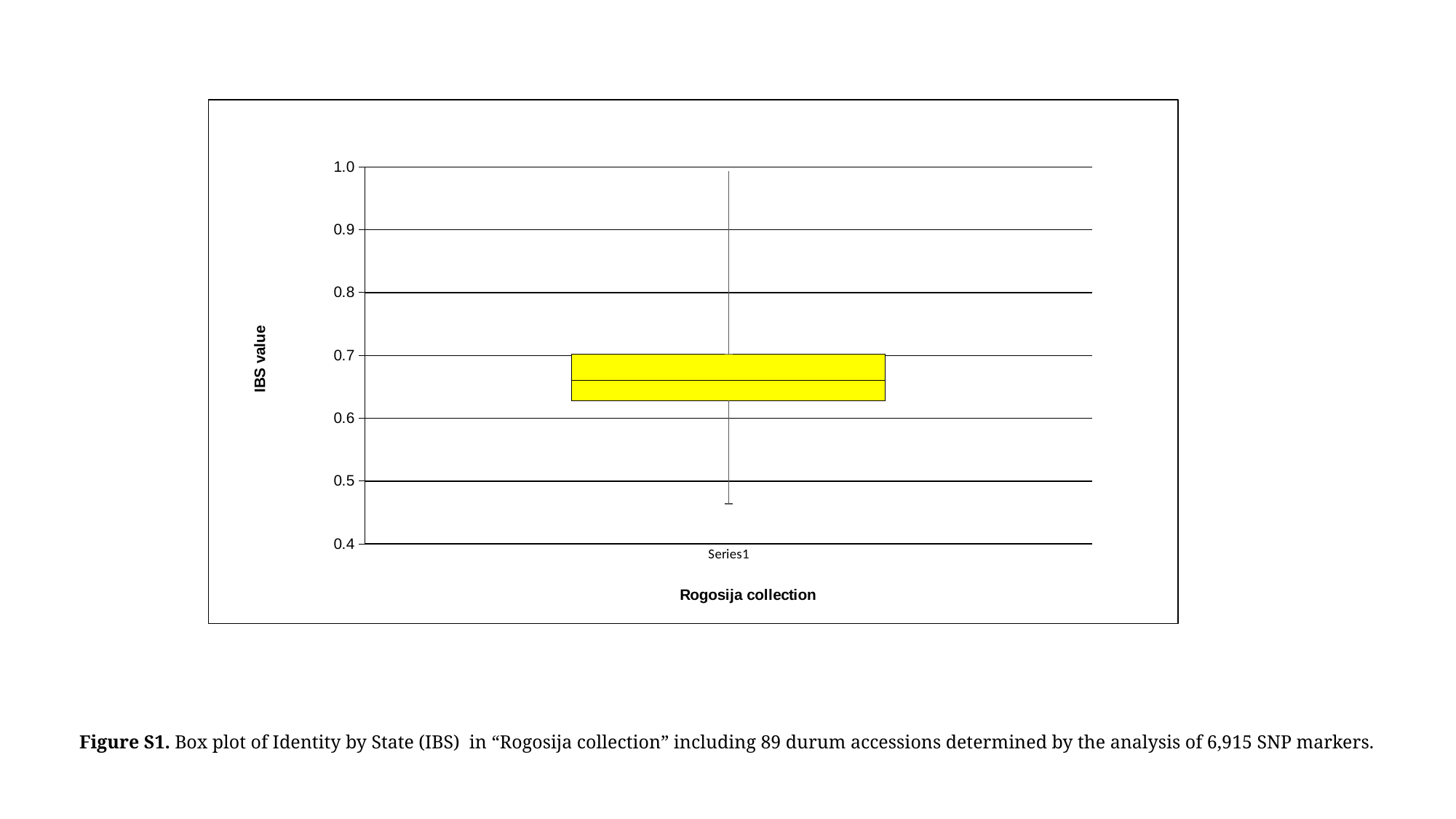

### Chart
| Category | | | | | |
|---|---|---|---|---|---|
| | 0.4637755 | 0.16427017750000006 | 0.032548587500000004 | 0.04127629999999993 | 0.291594436 |Figure S1. Box plot of Identity by State (IBS) in “Rogosija collection” including 89 durum accessions determined by the analysis of 6,915 SNP markers.

## Slide 2
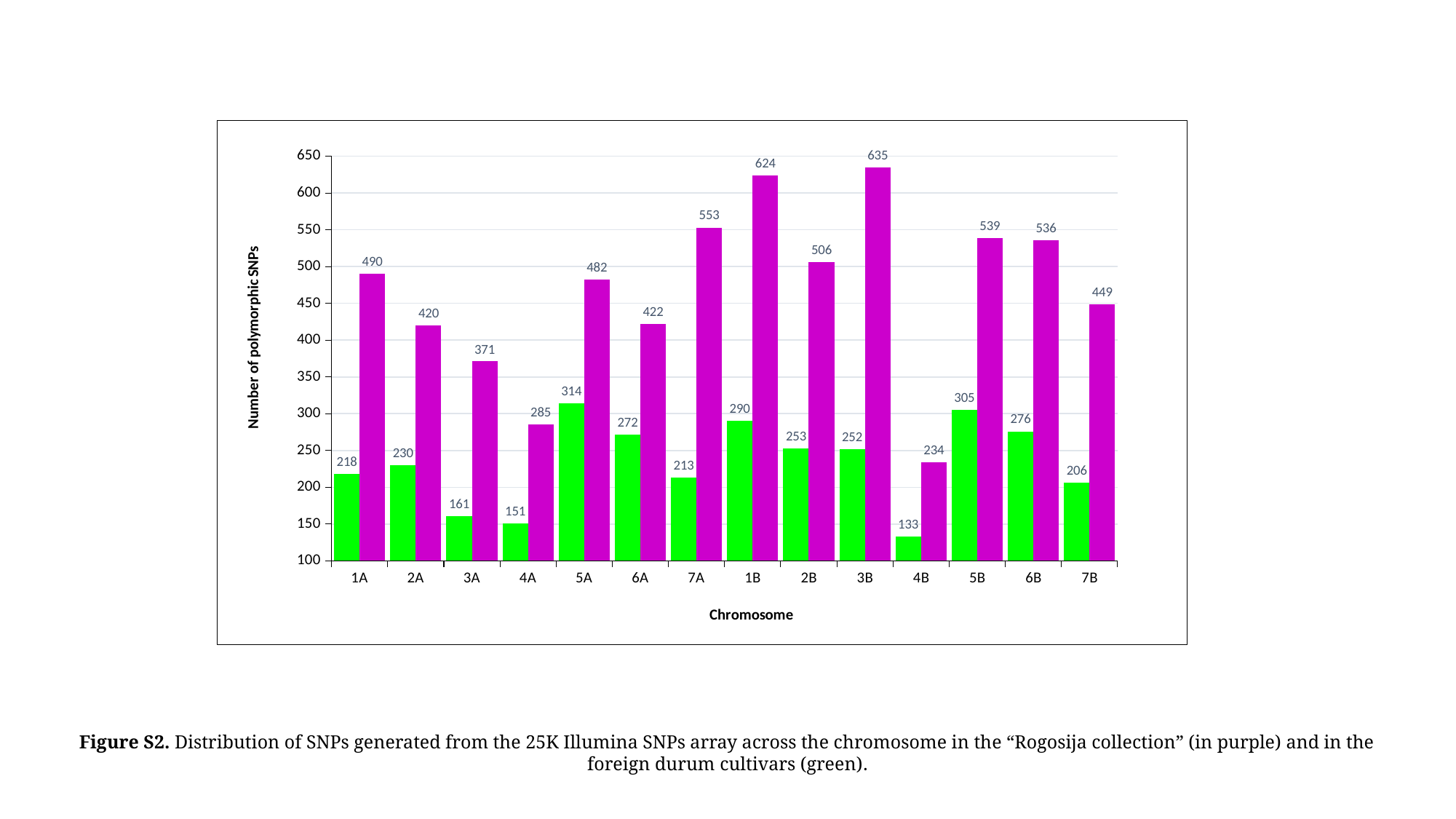

### Chart
| Category | | |
|---|---|---|
| 1A | 218.0 | 490.0 |
| 2A | 230.0 | 420.0 |
| 3A | 161.0 | 371.0 |
| 4A | 151.0 | 285.0 |
| 5A | 314.0 | 482.0 |
| 6A | 272.0 | 422.0 |
| 7A | 213.0 | 553.0 |
| 1B | 290.0 | 624.0 |
| 2B | 253.0 | 506.0 |
| 3B | 252.0 | 635.0 |
| 4B | 133.0 | 234.0 |
| 5B | 305.0 | 539.0 |
| 6B | 276.0 | 536.0 |
| 7B | 206.0 | 449.0 |Figure S2. Distribution of SNPs generated from the 25K Illumina SNPs array across the chromosome in the “Rogosija collection” (in purple) and in the foreign durum cultivars (green).

## Slide 3
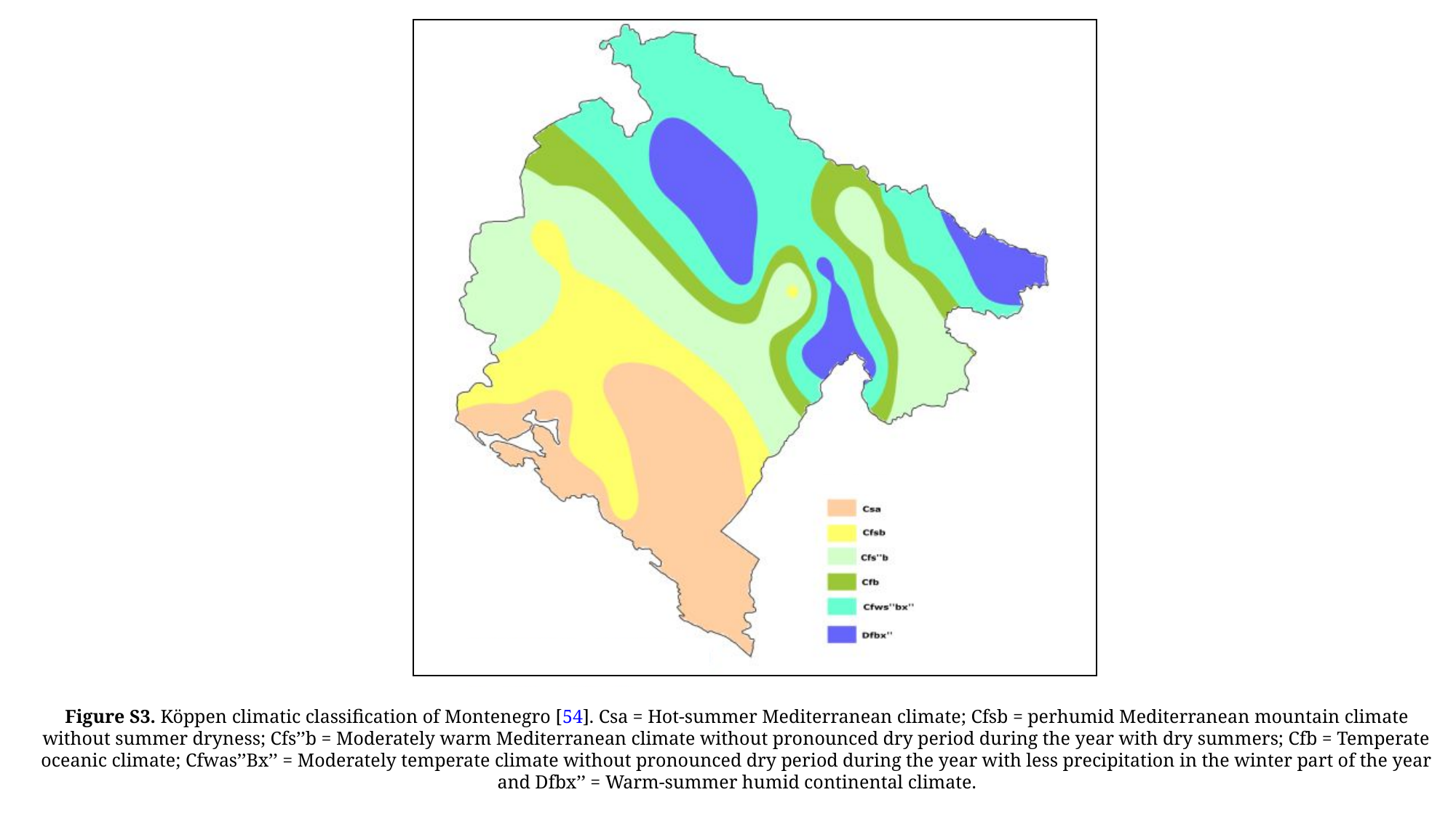

Figure S3. Köppen climatic classification of Montenegro [54]. Csa = Hot-summer Mediterranean climate; Cfsb = perhumid Mediterranean mountain climate without summer dryness; Cfs’’b = Moderately warm Mediterranean climate without pronounced dry period during the year with dry summers; Cfb = Temperate oceanic climate; Cfwas’’Bx’’ = Moderately temperate climate without pronounced dry period during the year with less precipitation in the winter part of the year and Dfbx’’ = Warm-summer humid continental climate.
